# Comment to: Factors affecting SARS-CoV-2 transmission among face mask users

**DOI:** 10.31744/einstein_journal/2025CE1532

**Published:** 2025-02-07

**Authors:** Camilla Mattiuzzi, Giuseppe Lippi

**Affiliations:** 1 Rovereto Hospital Provincial Agency for Social and Sanitary Services Trento Italy Medical Direction, Rovereto Hospital, Provincial Agency for Social and Sanitary Services, Trento, Italy.; 2 University of Verona School of Medicine Section of Clinical Biochemistry Verona Italy Section of Clinical Biochemistry, School of Medicine, University of Verona, Verona, Italy.

Dear Editor,

We read with interest the article by Costa et al.,^(1)^ which demonstrated that individuals who consistently wear face masks have a lower likelihood of carrying a high viral load after contracting severe acute respiratory coronavirus 2 (SARS-CoV-2) infection compared to those who do not regularly wear masks. This finding strongly supports the recommendation of face mask usage during periods of high viral circulation in outdoor crowded places, particularly among individuals at higher risk of developing more aggressive forms of coronavirus disease 2019 (COVID-19), such as frail and immunocompromised people. However, some methodological considerations merit attention.

First, the authors categorized participants as "regular" or "not regular" mask users based on reported time spent wearing masks, with "regular" users defined as those who wore masks "every time" or "most of the time." This classification lacks specificity and introduces potential ambiguity, as "most of the time" may overlap with "sometimes." A more precise approach would have been to quantify mask usage as a percentage of time spent outdoor, providing a clearer distinction between categories.

Second, the study did not account for the type of face masks used, a critical limitation given the varying efficacy of different mask types in preventing SARS-CoV-2 infection. As noted by the authors, this omission is significant. Evidence from a systematic review and meta-analysis by Kim et al. underscores this aspect.^(2)^ Specifically, medical or surgical masks were associated with a 29% reduction in SARS-CoV-2 infection risk, displaying an odds ratio (OR) of 0.71 and a 95% confidence interval (95% CI) of 0.44–1.14, while N95 respirators showed a 70% reduction in SARS-CoV-2 infection risk (OR= 0.30; 95%CI= 0.17–0.50). Notably, N95 respirators were over twice as effective as surgical masks, and the lower boundary of the 95%CI for surgical masks exceeded 1, raising concerns about their efficacy under certain conditions.

Finally, the study did not consider the time participants spent in crowded environments, a critical variable influencing SARS-CoV-2 infection risk. Studies, such as that by Garcia et al.,^(3)^ have shown that extended time in crowded settings-such as outdoor markets or public transportation hubs-correlates with higher SARS-CoV-2 transmission rates. It is plausible that individuals categorized as "not regular" mask users may have avoided prolonged exposure in such environments, potentially confounding the results. Adjusting for this factor would enhance the robustness of the study's conclusions.

In summary, while the findings by Costa et al.^(1)^ provide compelling evidence in favor of face mask use, addressing these methodological gaps would improve the accuracy and applicability of their conclusions.
